# Croatian Translation and Initial Psychometric Validation of the Negative Behaviors in Health Care Questionnaire

**DOI:** 10.3390/nursrep15020069

**Published:** 2025-02-14

**Authors:** Vesna Bušac, Nikolina Kanceljak, Ana Žepina Puzić, Ivona Ljevak

**Affiliations:** 1 Department of Health Studies, Šibenik University of Applied Sciences, 22000 Šibenik, Croatia; ana.zepina_puzic@vus.hr; 2 Faculty of Health Studies, University of Mostar, 88000 Mostar, Bosnia and Herzegovina; ivona.ljevak@fzs.sum.ba; 3 Society for Psychological Assistance, 10000 Zagreb, Croatia; nikolina@dpp.hr; 4 Faculty of Health Sciences, University of Novo Mesto, 8000 Novo Mesto, Slovenia

**Keywords:** lateral aggression, vertical aggression, incivility, translation, validation, nursing

## Abstract

**Background/Objectives**: This cross-sectional study aimed to produce an adapted Croatian version of the Negative Behaviors in Health Care Questionnaire and to validate it. **Methods**: The process comprised the translation, cultural adaptation, and psychometric evaluation of the questionnaire. Clinical specialists and qualified bilingual speakers participated in both forward and backward translation. Face validity was tested. The survey’s original developer approved the final version. The reliability of the questionnaire was assessed using the test–retest method and Cronbach’s alpha coefficient. Exploratory and confirmatory factor analyses and assessments of divergent and convergent validity were conducted. The collected data were analyzed using SPSS 21.0 and R, program version 3.5.2., for Windows. **Results**: A five-factor structure was obtained and confirmed via CFA, although not all fit coefficients were satisfactory. The internal consistency reliability was 0.86 for the contributing factors and the seriousness of aggression, 0.79 for the use of aggression, 0.95 for the fear of retaliation, and 0.83 for the frequency of aggression; in total, α = 0.88. Test–retest reliability was moderate. All correlations were statistically significant, and the correlation was the highest for seriousness (0.754) and frequency of aggression (0.725) and the lowest for contributing factors (0.528). Test–retest reliability was satisfactory. Statistically significant differences were found when comparing respondents by gender, age, work experience, education, and hierarchical position. **Conclusions**: The adapted, translated, and validated survey provides a valuable tool for assessing lateral and vertical aggression between and towards nurses in terms of contributing factors, frequency, severity, uses of aggression, and fear of retaliation.

## 1. Introduction

Workplace violence is a serious issue. It is widespread in healthcare settings, highlighting an urgent need for multi-stakeholder action [1]. Inadequate staffing, dysfunctional team dynamics, incompetent leadership, and strained nurse–physician relationships are all risk factors for violence in nursing workplaces [2]. In this context, violence includes physical assault, aggression [3], sexual harassment, bullying [4], verbal abuse [5], and threats, and can lead to injury, death, psychological harm, and mal-development. The global prevalence of non-physical violence among healthcare workers is estimated at 42.5% [6]. The causes of violence, in order of decreasing frequency, are patients, relatives, and peers. The terms most often used to describe negative behaviors among colleagues are workplace bullying, violence, aggression, abuse, hostility, sabotage, and incivility. Incidents of aggression between colleagues at the same hierarchical level are referred to as lateral (LA) or horizontal, and incidents between superiors and employees—which can happen in either direction but are more often directed downward—are referred to as vertical aggression (VA) [7]. These incidents are associated not only with lower productivity, employee retention, engagement, satisfaction, and increased absenteeism, but also with patient outcomes, treatment delays, medical errors, patient falls, and mortality [8,9]. According to some authors, they pose an iatrogenic risk to team work and patient safety [10,11,12].

Nurses who have experienced horizontal violence are more likely to leave or change careers [13,14]. LA and VA refer to undesirable behaviors towards colleagues of the same or different levels of power that cause psychological pain [8]. It is necessary to investigate specific sources of incivility and identify the knowledge and skills essential to maintaining a positive work environment, along with developing, applying, and evaluating effective educational and preventive steps [15]. Organizational and ergonomic strategies that lower stress and improve employee support and productivity should be part of the fight against workplace violence [9].

Inter-nurse LA can be influenced by hospital management, perpetrators, victims, and sociodemographic factors [16]. The research results emphasize the importance of systemic factors, such as stress at work and abuse of power, in shaping aggressive behavior in the healthcare system [17]. Involving department heads and management in reducing these behaviors can be crucial [18]. When the nursing environment is unfavorable, turnover may increase. Approximately 3.7 million nurses work abroad due to an adverse working environment in their home country. A shortage of 5.7 million nurses is predicted by 2030 [19]. In research on motives for leaving the profession, Kurtović et al. emphasize the urgent need to address burnout in young nurses. It would be interesting to investigate how negative behaviors in healthcare contribute to these findings [20].

To the authors’ knowledge, there is a lack of research on and a validated measurement instrument in the Croatian language for violence between healthcare professionals and the occurrence and characteristics of LA and VA. Although the problem has had global recognition for several decades, LA and VA are rarely mentioned in Croatia. A lack of confrontation and reporting does not mean that the problem does not exist. Potentially related topics in the Croatian language are violence against nurses, burnout, and mobbing [21]. These themes were occasionally mentioned in a review paper, as an integral part of other research in the form of individual questions or brief information, and in a graduate thesis [22,23,24]. This research area has not been systematically included in Croatian curricula for nursing education. If it is not presented as a separate course, it could be incorporated into courses on health psychology, communication skills, teamwork in nursing, etc. Except for those teaching classes on teamwork in nursing, course lecturers themselves are often not from the field of nursing. The education and healthcare systems do not prepare enough future graduates to respond to violent behavior. Newly employed nurses encounter this phenomenon, which is difficult to explain and handle. In addition to the challenges of mastering new tasks in stressful situations of working under extreme pressure, shift work, and adapting to a new work environment, new nurses are often unable to perceive or adequately react to instances of violence. Violent behavior mostly goes unpunished; if the victim resists, it is possible that they will be re-victimized. There are no developed reporting or support systems. The general public and witnesses of the event remain silent. Legislative support persists on paper but is rarely implemented. Turning a blind eye to inappropriate behavior among and towards nurses is common. These incidents are considered normal and a path of initiation into the profession. Many nurses and technicians give up on this path. The translation and validation of the Negative Behaviors in Health Care (NBHC) questionnaire should be a small step towards addressing the painful topic of inappropriate behavior towards and between nurses within the healthcare system, including its frequency, forms, and severity [25]. In a national survey conducted by the Croatian Chamber of Nurses in 2018, 40% of nurses stated that they had experienced some form of violence by other healthcare workers. Very few respondents reported violence. Violence occurred in 73% of the institutions examined by the study [26]. Furthermore, according to a multinational study of intensive care unit nurses from Croatia, Poland, Spain, Cyprus, and Romania, the most violence towards nurses came from patients (n = 2050), followed by other nurses (n = 1453) and doctors (n = 1039) [23]. This cross-sectional study aims to translate, adapt, and validate the NBHC survey for use in Croatia. This could be a starting point for collecting data to draw concrete conclusions about the frequency, forms, and severity of the consequences of LA and VA in this area, and for comparing differing cultural contexts and methodological approaches among similar studies.

## 2. Materials and Methods

This cross-sectional psychometric study evaluated the cultural compatibility and psychometrics of the NBHC questionnaire among Croatian nurses. It comprised questionnaire translation and validation. Translation was carried out according to Polit and Yang’s guidelines [27]. For ethical considerations, permission to translate and validate the NBHC survey was obtained from the original designers of the questionnaire D.M. Layne et al. from Medical University of South Carolina, Charleston, SC, USA [25].

### 2.1. Translation

**Figure 1:**
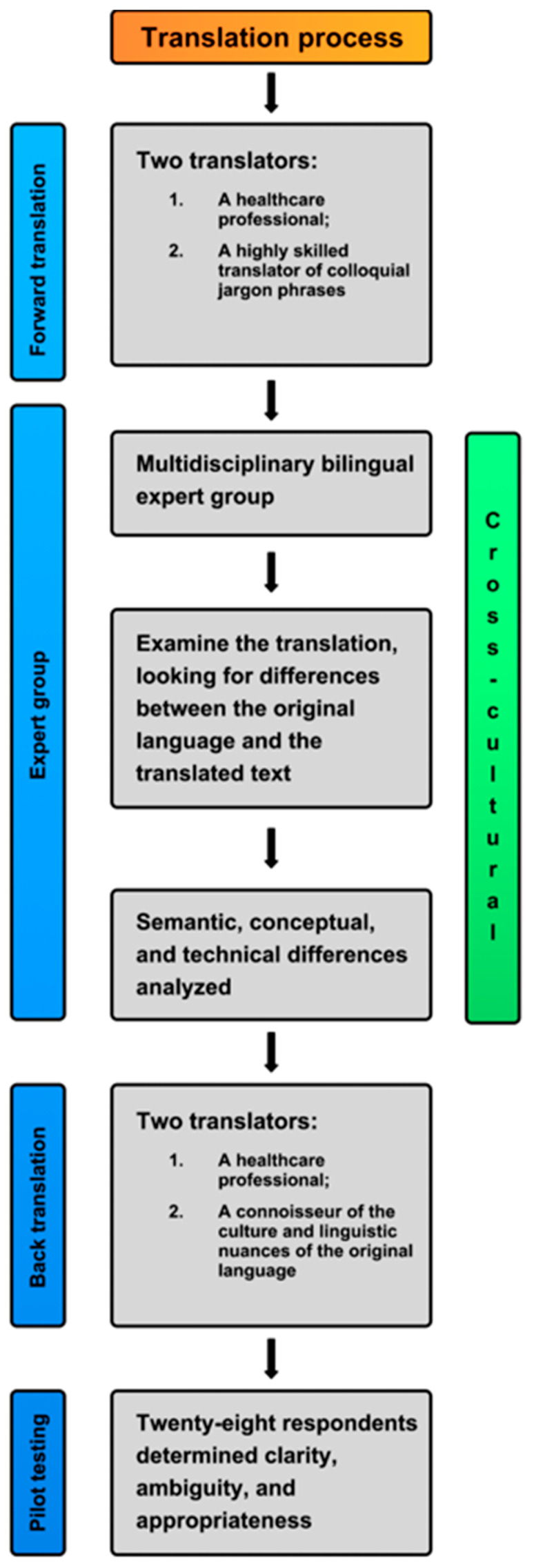
Negative Behaviors in Health Care (NBHC) questionnaire translation and cross-cultural adaptation process.

Forward and backward translation was conducted, with two translators participating in each process. The first two translators were highly fluent in and familiar with both languages and cultures, and the target language was their mother tongue. One was a health professional, while the other was an excellent translator of colloquial jargon phrases who was unfamiliar with the actual construction of the questionnaire and medical language. They independently translated the questionnaire into Croatian. A multidisciplinary bilingual expert group was formed to examine the translation of each part, looking for differences between the original language and the translated text. This group consisted of experts from nursing, medicine, and psychology, as well as English- and Croatian-language teachers. Semantic, conceptual, and technical inequities were considered with the aim of preserving the integrity of the original instrument. In terms of cultural adaptation, the expert group discussed several concepts and statements and decided to harmonize the questionnaire’s content with the spirit of the Croatian language (Table 1). Two other bilingual and bicultural translators back-translated the questionnaire into English without having read the original questionnaire. One was a health professional, while the other was a connoisseur of the culture and linguistic nuances of the original language. After the experts agreed on the final version, the questionnaire was sent to the author for approval (Figure 1).

### 2.2. Pilot Testing

This phase was included to guarantee that all translated items were clear, easy to understand, and unambiguous. Pilot testing was conducted on a small group of respondents with characteristics similar to those in the final questionnaire. Nurses who were not included in the test–retest phase participated. Twenty-eight respondents determined the questionnaire’s clarity, ambiguity, and appropriateness by rating each part with a value of 0 or 1 if the translation was inadequate or adequate, respectively. Krippendorff’s alpha test was used to assess inter-rater reliability for various items. The results show high reliability (1.00) for all items, indicating consistency among raters (Figure 1). 

### 2.3. Test–Retest

The questionnaire was tested and retested. To ensure participant anonymity after the test phase and adequately organize the collected data, each participant independently created a unique code when completing the questionnaire. The initial questionnaire required 10–15 min, while the retest required only 5 min. Sociodemographic information and the questionnaires used to evaluate convergent and divergent validity during the test phase were not included in the retest phase. In the retest phase, 112 of the same respondents participated. Two months passed between the test and retest data collection periods.

### 2.4. Measuring Instrument

The 25 items of the NBHC were split into five subscales: the use of aggression, the fear of retaliation, the frequency of aggression, the seriousness of aggression, and the contributing factor. Unlike the original questionnaire, which used a four-point Likert scale in three subscales, the adapted version used a five-point Likert scale in all subscales [25]. A nine-item subscale measured factors that contribute to lateral aggression (LA) and vertical aggression (VA). Seven items assessed the frequency of aggression, six assessed the severity of aggression, and three examined the fear of retaliation. Two optional open-ended questions asked the participants to describe their LA and VA experiences and provide recommendations that could help lower the occurrence of violence. The participants submitted the ten sociodemographic questions throughout the test phase. In the test phase, the Toronto Empathy Questionnaire [28] and the Aggression Questionnaire [29] were used in addition to the NBHC questionnaire to assess divergent and convergent validity, with the authors’ permission.

### 2.5. Sample

The sample size was calculated according to Field’s instructions from 2013, which recommended 5–10 respondents per questionnaire item [30]. A total of 193 participants took part in the test study; most were female (n = 172, 89.12%), with an average age of 36.01 years (SD = 12,452, min = 20, max = 68). Most participants came from urban areas (n = 136, 71.1%), and a smaller number came from rural areas (n = 57, 28.9%). The largest number of participants had completed high school (n = 108, 55.9%), while the smallest had completed graduate study (n = 36, 18.7%). The average number of years of experience in the profession was 14.78 (SD = 12.182); the participants’ length of work experience ranged from 5 months to 43 years.

### 2.6. Data Collection

A random sample of respondents were provided an online form by the institution’s management. The inclusion criterion was a valid work license. Participation was voluntary and could be withdrawn at any time. The purpose, goal, and questionnaire-filling method were described. Respondents provided consent to participate in the study. Data were collected from April to June 2024.

### 2.7. Data Analysis

There were no missing data, as all responses were mandatory except for two open-ended questions, which were not analyzed in this study. Data were collected and stored using Microsoft Excel (version 11, Microsoft Corporation, Redmond, WA, USA) and analyzed with SPSS 21.0 (IBM Corp., Armonk, NY, USA) and R software for Windows version number 3.5.2. (Lavaan package). Descriptive statistics (means, standard deviations, and score ranges) were calculated for all variables. Krippendorff’s alpha test was used to assess inter-rater reliability for various items. The Shapiro–Wilk test was used to assess the normality of the distribution. The dimensionality of the questionnaire was tested using exploratory factor analysis (EFA), with the principal component method and varimax rotation. The internal consistency of the instrument was assessed using Cronbach’s alpha coefficient (α), while test–retest reliability was evaluated using Spearman’s correlation coefficient. To determine the appropriate correlation method for (con)divergent validity, the Shapiro–Wilk test results guided the selection of Spearman’s correlation coefficients, as most subscales significantly deviated from normality. CFA was carried out using the Lavaan package in R to validate the factor structure. Model fit was evaluated using the following indices: Comparative Fit Index (CFI), Root Mean Square Error of Approximation (RMSEA), and Standardized Root Mean Square Residual (SRMR). The Mann–Whitney U and Kruskal–Wallis tests were used to compare sociodemographic data.

### 2.8. Ethical Considerations

This study was conducted with strict adherence to ethical guidelines and principles to ensure the protection and rights of all participants. All participants provided informed consent before beginning the study. Since data collection was conducted online, the participants were first presented with an electronic consent form explaining the study’s purpose, the voluntary nature of their participation, and data confidentiality measures. Only after actively confirming their consent by selecting the ‘I agree’ option were they allowed to proceed with the questionnaire. Participants could withdraw at any time without consequence. E-mail addresses were collected in the test phase so that participants could be contacted again for the retest phase. The contact information was archived separately from the research results; only one researcher had access. The results were coded to connect the data from the test and retest phases. Ethical approval was obtained from the relevant institutional ethics committees before data collection (Class: 007-10/24-01/7; File Number: 24-2) and (Class: 004-05/24-01/06; File Number: 2182-10-17/09-24-3). Only the research team had access to the securely stored data. Permission was also obtained from the authors of the original questionnaires. This study followed the principles of the 1964 Declaration of Helsinki (2013 revision).

## 3. Results

### 3.1. Exploratory Factor Analysis

The dimensionality of the questionnaire was tested on this sample using an exploratory factor analysis under the principal components model. Bartlett’s test determined the correlation matrix and its significance, and the suitability of the correlation matrix for factorization was determined using the Kaiser–Meyer–Olkin sampling adequacy test. Bartlett’s test of the correlation matrix’s significance returned a high value (χ²= 3814.192), indicating significance with a risk of less than 1%. The Kaiser–Meyer–Olkin index of sampling adequacy was 0.86, which shows that the correlation matrix of the measuring instrument variables is suitable for implementing factorization. The factor analysis, according to the Gutman–Kaiser criterion with varimax rotation, showed that there are a total of five factors in the latent structure of the questionnaire, which explained a total of 66.28% of the variance and are differentiated on the scree plot. The overall reliability of the scale, measured using Cronbach’s alpha, was calculated. (Table 2).

### 3.2. Normality

The normality of the distribution of the results of contributing factors and the seriousness of aggression was tested and found to be positively asymmetric (shifted towards lower values). In contrast, the remaining factors were negatively asymmetric (Table 3).

### 3.3. Test–Retest Reliability

The questionnaire was applied to the same group of participants to check test–retest reliability. Since the distribution of results significantly deviated from normal on all individual factors, the non-parametric Spearman correlation test was applied to determine test–retest reliability (Table 4).

### 3.4. Confirmatory Factor Analysis

Confirmatory factor analysis, a statistically powerful procedure, helps test how well a specific theoretically based model fits empirical data. In this case, the five defined factors were checked to see if they corresponded to the collected results. The results of the confirmatory factor analysis are shown in Table 5.

### 3.5. Normality of Distribution for the Toronto Empathy Questionnaire and Subscales of the Aggression Questionnaire

The distribution of results on the empathy and anger scales does not deviate significantly from normal, while on the physical aggression, hostility, and verbal aggression subscales, it deviates significantly from normal—it is positively asymmetric, i.e., shifted towards lower values (Table 6).

The distribution of results on the empathy and anger scales does not deviate significantly from normal, while on the physical aggression, hostility, and verbal aggression subscales, it deviates significantly from normal—it is positively asymmetric, i.e., shifted towards lower values.

### 3.6. Divergent Validity

To check the questionnaire’s divergent validity, the Toronto Empathy Questionnaire was applied. Since the questionnaires measure different constructs that are not expected to have significant correlations, it was expected that there would be no significant correlations between individual factors of the questionnaire and the total result on the Toronto Empathy scale. Spearman’s correlation coefficient was used, and satisfactory divergent validity was determined (Table 7).

### 3.7. Convergent Validity

The Aggression Questionnaire was applied to check the convergent validity of the questionnaire; it was expected that its specific factors would be significantly correlated with the characteristics of the NBHC questionnaire. Factor analysis for the aggression scale showed the existence of four factors that explained 47.85% of the variance: physical aggression (9 items), anger (9 items), hostility (5 items), and verbal aggression (4 items) (Table 8).

### 3.8. Comparison of Sociodemographic Variables

Significant differences with respect to gender were found for factors 1 (*p* = 0.004) and 5 (*p* = 0.045), with male participants achieving higher scores on both factors compared to females (Table 9).

Regarding differences by environment—urban vs. rural—no statistically significant differences were found in the results on individual factors of the scale between those who came from urban areas and those who came from rural areas.

In the Mann–Whitney test, a significant difference with respect to the hierarchical position in the organization was determined only for factor 3 (*p* = 0.045), where employees achieved higher results on the scale compared to team leaders (Table 10).

For differences according to work schedule, no statistically significant differences were found in the results on individual factors with respect to the participants’ work schedules.

The Kruskal–Wallis test identified statistically significant differences in the results for factor 1 (*p* = 0.004) with respect to the age of the participants, with the youngest employees (up to 30 years of age) achieving the highest results on the scale, while the lowest results for this factor were achieved by employees aged between 31 and 40 (Table 11).

The Kruskal–Wallis test determined statistically significant differences in the results for factor 1 (*p* = 0.001) with respect to the level of education, with employees with only a high school education achieving the highest results on the scale, while employees with a graduate degree achieved the lowest results (Table 12).

The Kruskal–Wallis test revealed statistically significant differences in the results for factor 1 (*p* = 0.046) with regard to the length of service in the profession, whereby employees with the shortest service (up to 5 years) achieved the highest results on the scale, while the lowest results were achieved by employees with the most seniority (over 15 years) (Table 13).

## 4. Discussion

This research provides valuable insight into the dimensionality, reliability, and validity of the adopted NBHC questionnaire, with results that prompt further discussion and consideration. The target population in this study was nurses. As nurses are still predominantly female, the stratification of the sample by gender reveals domination by women (89.12%); 70.5% were from urban environments, the majority of which have a high school education level (55.9%).

In an integrative review of instruments measuring negative health behaviors, Layne et al. identified 22 measurement instruments with acceptable psychometric properties. Only two questionnaires underwent test–retest and validity assessments. Five questionnaires demonstrated construct validity and convergent validity; four instruments reported face validity. Cronbach’s alpha ranged from 0.77 to 0.99. Most responses were provided via a Likert scale from 1 to 4 or 5. Participants included managers, colleagues, physicians, patients, or families. The questionnaires most often measured the behavior between nurses, rarely measuring behavior among multidisciplinary teams or teams of health professionals. In similar questionnaires, the psychometric properties of the final instrument were often neglected.

Key principles for measuring negative behaviors were defined as the frequency, severity, and source of behavior. Most existing instruments measure only one or two principles, and it was concluded that this type of questionnaire must be improved by conducting research in various environments and creating a factor analysis, which has not been completed for the majority of studies, as well as a test–retest phase for examining reliability within the sampled population. It is necessary to strengthen awareness of the benefits of using validated questionnaires, standardizing terminology, and improving existing measurement tools without creating a multitude of unfinished attempts [31].

The original questionnaire in the subscales contributing factors, severity of aggression, and fear of retaliation, had four response options on a Likert scale [25]. In the translated and adapted NBHC survey, a five-point Likert scale was developed for the same subscales to enable more nuanced answers and precisely expressed attitudes and opinions [32]. This study’s exploratory factor analysis revealed a five-factor structure with high internal consistency and reliability. The reliability of each of the five factors has extremely high internal consistency for α = 0.88 in total, and the distribution of items by factors is completely identical to that obtained in the validation study by Layne et al. [25]. For the five factors, Cronbach’s alpha coefficient was as follows: contributing factors, 0.86; seriousness of aggression and use of aggression, 0.79; fear of retaliation, 0.95; and frequency of aggression, 0.83. However, test–retest reliability was moderate. All correlations were statistically significant. The correlation was the highest for the factor’s seriousness (0.754) and the frequency of aggression (0.725), while it was the lowest for contributing factors (0.528). These results indicate the possibility that the participants’ perceptions of this factor varied over time, which may be a consequence of changing working conditions or personal circumstances. The results indicate moderate relationships between theoretically related constructs. However, since the observed convergent validity coefficients were below the commonly accepted threshold of 0.6, the findings provide only partial support for convergent validity. This suggests that while the constructs are related, additional validation may be required to confirm stronger associations. Future research should consider alternative measurement approaches or an extended sample to further investigate the scale’s construct validity. Negative correlations between aggression and dimensions such as frequency and severity of aggression further support the instrument’s validity [33]. Although the five-factor structure was confirmed, the fit indices of the model (CFI = 0.811; RMSEA = 0.114; SRMR = 0.197) suggest a need for further optimization. In the future, it would be desirable to consider expanding and stratifying the sample and analyzing the model between groups to improve the CFI. In the literature, various criteria for index values show whether the structure of the measuring instrument is satisfactory. It is recommended that the CFI should not be higher than 0.90 [34]; in this case, it was 0.811. The RMSEA and SRMR indices should be less than 0.10 [35,36], but these indices had slightly higher values in this study. A relative Chi-square value less than 3.00 is most often accepted as a model of good fit to the data, although in practice, some researchers also accept a value of 5.00 [37]. In this case, it was 5.028. Confirmatory factor analysis thus confirmed the five-factor structure of the questionnaire to some extent.

Various studies provide an overview of the influence of the respondents’ demographic characteristics on reporting violent behavior, as well as lateral and vertical aggression. This analysis of sociodemographic parameters reveals several significant differences among respondents based on their characteristics. These results provide valuable insights into how sociodemographic factors can influence the emotional and aggressive responses of individuals within organizations.

Gender Differences: This study identified significant differences in scores between male and female participants for factor 1 (*p* = 0.004) and factor 5 (*p* = 0.045). Male participants scored higher on both factors compared to females, which may suggest differences in emotional or aggressive responses between genders. These findings indicate a possible gender-based divergence in how aggression is expressed or perceived, aligning with previous research on lateral and vertical aggression. Such results underscore the need for further investigation into gender-specific interventions to address workplace aggression effectively [38].

Differences by Level of Education: The Kruskal–Wallis test showed significant differences (*p* = 0.001) based on education level, with employees who completed high school scoring the highest, while those with a diploma had the lowest scores. This finding suggests that education level may influence emotional or aggressive responses [39]. A 2023 integrative review provides important insights into experiences of inappropriate behavior among recently graduated nurses. The review highlights temporary employment as a key factor contributing to increased exposure to such behavior. Similarly, the impact of rudeness toward nursing students has been discussed in the literature, further emphasizing the need for supportive learning and professional environments [40].

Differences by Hierarchical Position: A notable finding of this study is that nurse leaders sometimes exhibit a lack of knowledge of interprofessional violence, which can influence their ability to effectively address such issues within the organization. Our results reveal a significant difference in experiences of lateral and vertical aggression based on hierarchical position, with employees scoring higher than team leaders for factor 3 (*p* = 0.045). This suggests that perceptions and responses to workplace aggression may vary significantly depending on one’s position within the organizational hierarchy [39]. These findings emphasize the importance of targeted education and training for nurse leaders to improve their understanding and management of interprofessional violence.

Differences by Age: The Kruskal–Wallis test revealed significant differences for factor 1 (*p* = 0.004) based on participants’ age. The youngest employees (under 30 years-old) had the highest scores, while those aged 31 to 40 had the lowest. This may suggest that emotional intelligence or aggression levels change with age [17,41,42].

Differences by Length of Service: Significant differences were also found for factor 1 (*p* = 0.046) based on length of service. Employees with the shortest tenure (up to 5 years) had the highest scores, while those with the longest tenure (over 15 years) had the lowest. This trend may reflect changes in attitudes or behaviors over the course of a career [41].

Differences by Environment: No statistically significant differences were found between participants from urban and rural areas. This suggests that the environment may not play a key role in the measured variables, which contradicts the findings of Mohammad et al. [38].

Differences by Work Schedule: No significant differences were found in the results based on participants’ work schedules, indicating that work mode does not impact the variables analyzed.

## 5. Strengths and Limitations of the Study

This study has several strengths. It represents the first Croatian translation and psychometric validation of the Negative Health Behaviors Questionnaire, ensuring cultural and linguistic adaptation. The study uses comprehensive statistical analyses, including exploratory and confirmatory factor analyses, test–retest reliability, and convergent and divergent validity assessments. Additionally, the instrument’s high internal consistency supports its reliability for measuring negative behaviors. It is applicable to all healthcare professionals.

However, some limitations must be acknowledged. With a random sampling of respondents, the questionnaire is applicable to individuals with a healthcare license, but does not have the ability to survey, for example, students. In addition, since the study relies on self-assessment data, response bias may be present due to social desirability or recall limitations, especially in items that address aggression towards others.

Future research should aim to validate the instrument in a broader sample, including other health professions and settings.

## 6. Conclusions

This study provides a foundation for further research into negative behavior regarding LA and VA in healthcare institutions. The questionnaire’s identified dimensions can help develop targeted interventions to reduce aggressive behavior and create a safer work environment. The practical implications of translating and validating the NBHC questionnaire into Croatian include the possibility of measuring the systematic assessment of lateral and vertical aggression in healthcare in the Republic of Croatia and countries with similar languages and cultures. These results indicate that the NBHC questionnaire is a valid and reliable instrument, with high internal consistency, satisfactory test–retest reliability, and an acceptable factor structure. This research provides a solid foundation for further applying the NBHC questionnaire. Data obtained with this new instrument can be used to develop targeted educational programs and interventions to improve working relations and reduce conflicts. The instrument also enables monitoring the frequency and causes of negative behavior over time, supporting strategic decision-making in human resource management. A validated questionnaire can strengthen the professional status of nurses and create a safer and more supportive work environment.

Further instrument validation and implementation in different contexts are crucial for practical application. To verify and improve these findings, this study recommends repeating the research with a larger sample and possibly improving the CFI model.

## Figures and Tables

**Table 1:** Cultural adaptation of the translation.

Original NBHC Survey	Cultural Adaptation to Croatian
Negative behaviors	Neprimjerena ponašanja
Contributing factors	Pogodujući čimbenici
Power and control issues	Zlouporaba moći i želja za kontrolom
Major personality clashes	Sukobi zbog velikih karakternih razlika
I feel safe from retaliation when reporting an episode of lateral aggression	Ne osjećam strah od osvete kada prijavim epizodu vodoravne agresije
I feel safe from retaliation when reporting an episode of lateral aggression	Ne osjećam strah od osvete kada prijavim epizodu vodoravne agresije
I feel safe from retaliation when reporting an episode of vertical aggression directed downward	Ne osjećam strah od osvete kada prijavim epizodu okomite agresije usmjerene prema podređenima
I feel safe from retaliation when reporting an episode of vertical aggression directed upward	Ne osjećam strah od osvete kada prijavim epizodu okomite agresije usmjerene prema nadređenima

**Table 2:** Factor saturations obtained via exploratory factor analysis using the principal component model with varimax rotation (n = 193).

	Contributing Factors	Seriousness	Uses Aggression	Fear of Retaliation	Frequency of Aggression
Rude behavior	0.726				
Leaders not willing to intervene	0.782				
Peers not willing to intervene	0.767				
The targeted person not willing to stand up to the perpetrator	0.759				
Major personality clashes	0.677				
Job stress leading to loss of control over behavior	0.617				
Inadequate staff/resources to handle the workload	0.585				
Misunderstandings related to cultural differences	0.478				
I am the recipient of vertical aggression directed downward					0.682
I observe lateral aggression					0.668
I am the recipient of lateral aggression					0.649
I use vertical aggression directed downward			0.963		
I observe vertical aggression directed downward from healthcare professionals in leadership positions					0.627
I use vertical aggression directed upwards			0.852		
I use lateral aggression			0.800		
Compared to other workplace stressors, lateral aggression is		0.902			
Vertical aggression directed upward		0.875			
Compared to other workplace stressors, vertical aggression is		0.873			
Vertical aggression directed downward		0.863			
Lateral aggression toward new healthcare professionals		0.831			
Lateral aggression toward healthcare professional peers		0.475			
I feel safe from retaliation when reporting an episode of vertical aggression directed downward				0.932	
I feel safe from retaliation when reporting an episode of vertical aggression directed upward				0.874	
I feel safe from retaliation when reporting an episode of lateral aggression				0.862	
**Eigen value**	8.14	3.21	3.06	2.38	1.24
**% Explained variance**	31.25	11.74	11.35	8.44	3.51
**Cronbach’s alpha**	0.91	0.90	0.92	0.95	0.83
**Total cronbach’s alpha**		0.88			

**Table 3:** Testing the normality of the distribution of results on the 5 factors of the questionnaire with the Shapiro–Wilk test.

	S-W	df	*p*
f1	0.950	193	<0.0001 **
f2	0.923	193	<0.0001 **
f3	0.416	193	<0.0001 **
f4	0.940	193	<0.0001 **
f5	0.950	193	<0.0001 **

** *p* < 0.001.

**Table 4:** Test–retest reliability assessed using the Spearman correlation coefficient (n = 112).

	f1–1. Measurement	f2–2. Measurement	f3–3. Measurement	f4–4. Measurement	f5–5. Measurement
f1–1. Measurement	0.528 **				
f2–2. Measurement		0.754 **			
f3–3. Measurement			0.606 **		
f4–4. Measurement				0.549 **	
f5–5. Measurement					0.725 **

** *p* < 0.001.

**Table 5:** Fit indices for the 5-factor model of the Inappropriate Behavior Questionnaire.

	Index Values
Relative Chi-square	5.028
CFI	0.811
RMSEA	0.114
SRMR	0.197

**Table 6:** Normality of distribution for the Toronto Empathy Questionnaire and subscales of the Aggression Questionnaire.

	Shapiro–Wilk		
	Statistics	df	Sig.
Empathy	0.992	193	0.375
Physical aggression	0.824	193	<0.001 **
Anger	0.990	193	0.182
Hostility	0.970	193	<0.001 **
Verbal aggression	0.983	193	0.020 *

* *p* < 0.05, ** *p* < 0.001.

**Table 7:** Testing correlations between 5 factors and the total score on the empathy scale, using Spearman’s correlation coefficient.

	Empathy
f1a	0.043 *
f2a	0.077
f3a	0.062
f4a	−0.063
f5a	<0.001 **

* *p* < 0.05, ** *p* < 0.001.

**Table 8:** Spearman’s correlation coefficients for five factors of the questionnaire and subscales of the Aggression Questionnaire.

	Physical Aggression	Anger	Hostility	Verbal Aggression
Contributing factors	0.003	−0.099	−0.023	−0.001
Seriousness	−0.142 *	−0.162 *	−0.010	−0.048
Uses Aggression	−0.225 **	−0.223 **	−0.206 **	0.034
Fear of Retaliation	−0.033	0.041	0.127	−0.113
Frequency of Aggression	−0.083	−0.241 **	−0.246 **	0.005

* *p* < 0.05, ** *p* < 0.001.

**Table 9:** Gender differences among factors: NBHC.

Factor	Gender	Mean Rank	Mann–Whitney U	Z	*p*-Value (Asymp. Sig.)
F1	Female	92.95	1109.000	−2.887	0.004 **
	Male	130.19			
F2	Female	95.00	1461.500	−1.430	0.153
	Male	113.40			
F3	Female	97.34	1747.000	−0.338	0.735
	Male	94.19			
F4	Female	97.81	1667.500	−0.580	0.562
	Male	90.40			
F5	Female	94.19	1323.000	−2.008	0.045 *
	Male	120.00			

* *p* < 0.05, ** *p* < 0.001.

**Table 10:** Differences with respect to position–hierarchy.

Factor	Position	Mean Rank	Mann–Whitney U	Z	*p*-value (Asymp. Sig.)
F1	Employee	99.68	1867.500	−1.621	0.105
	Team leader	81.20			
F2	Employee	98.62	042.500	−0.982	0.326
	Team leader	87.45			
F3	Employee	99.39	1915.500	−2.001	0.045 *
	Team leader	82.91			
F4	Employee	96.22	2180.500	−0.479	0.632
	Team leader	101.63			
F5	Employee	98.28	2098.000	−0.779	0.436
	Team leader	89.43			

* *p* < 0.05.

**Table 11:** Differences with respect to participant age.

Factor	Age Group	Mean Rank	Chi-Square	*p*-Value (Asymp. Sig.)
F1	18–30	112.10	10.912	0.004 **
	31–40	84.29		
	41+	85.94		
F2	18–30	96.03	0.561	0.755
	31–40	92.51		
	41+	100.55		
F3	18–30	101.95	2.242	0.326
	31–40	93.08		
	41+	93.25		
F4	18–30	90.23	2.270	0.321
	31–40	100.80		
	41+	102.97		
F5	18–30	103.00	2.723	0.256
	31–40	85.13		
	41+	96.25		

** *p* < 0.001.

**Table 12:** Differences with respect to level of education.

Factor	Level of Education	Mean Rank	Chi-Square	*p*-Value (Asymp. Sig.)
F1	high school	109.26	13.083	0.001 **
	undergraduate study	87.36		
	graduate study	73.72		
F2	high school	102.61	2.519	0.284
	undergraduate study	90.76		
	graduate study	88.72		
F3	high school	97.16	0.911	0.634
	undergraduate study	100.45		
	graduate study	92.05		
F4	high school	96.68	1.594	0.451
	undergraduate study	90.78		
	graduate study	106.00		
F5	high school	104.64	5.433	0.066
	undergraduate study	92.04		
	graduate study	81.14		

** *p* < 0.001.

**Table 13:** Differences with respect to work experience.

Factor	Age Group	Mean Rank	Chi-Square	*p*-Value (Asymp. Sig.)
F1	18–30	112.10	10.912	0.004 *
	31–40	84.29		
	41+	85.94		
F2	18–30	96.03	0.561	0.755
	31–40	92.51		
	41+	100.55		
F3	18–30	101.95.	2.242	0.326
	31–40	93.08		
	41+	93.25		
F4	18–30	90.23	2.270	0.321
	31–40	100.80		
	41+	102.97		
F5	18–30	103.00	2.723	0.256
	31–40	85.13		
	41+	96.25		

* *p* < 0.05.

## Data Availability

The data presented in this study are available on request from the corresponding author.

## Supplementary Materials

The following supporting information can be downloaded at: https://www.mdpi.com/article/10.3390/nursrep15020069/s1, STROBE Statement—Checklist of items that should be included in reports of cross-sectional studies

## Public Involvement Statement

There was no public involvement in this research.

## Guidelines and Standards Statement

This manuscript was prepared following the procedures outlined in the Strengthening the Reporting of Observational Studies in Epidemiology (STROBE) Statement for observational studies in Supplementary Materials.

## References

[1] O’Brien C.J., Van Zundert A.A.J., Barach P.R. (2024). The growing burden of workplace violence against healthcare workers: Trends in prevalence, risk factors, consequences, and prevention—A narrative review. eClinicalMedicine.

[2] Havaei F., Astivia O.L.O., MacPhee M. (2020). The impact of workplace violence on medical-surgical nurses’ health outcome: A moderated mediation model of work environment conditions and burnout using secondary data. Int. J. Nurs. Stud..

[3] Zhang J., Zheng J., Cai Y., Zheng K., Liu X. (2021). Nurses’ experiences and support needs following workplace violence: A qualitative systematic review. J. Clin. Nurs..

[4] Shorey S., Wong P.Z.E. (2021). A qualitative systematic review on nurses’ experiences of workplace bullying and implications for nursing practice. J. Adv. Nurs..

[5] Mammen B.N., Lam L., Hills D. (2023). Newly qualified graduate nurses’ experiences of workplace incivility in healthcare settings: An integrative review. Nurse Educ. Pract..

[6] Liu J., Gan Y., Jiang H., Li L., Dwyer R., Lu K., Yan S., Sampson O., Xu H., Wang C. (2019). Prevalence of workplace violence against healthcare workers: A systematic review and meta-analysis. Occup. Environ. Med..

[7] Zapf D., Escartín J., Scheppa-Lahyani M., Einarsen S.V., Hoel H., Vartia M. (2020). Empirical findings on prevalence and risk groups of bullying in the workplace. Bullying and Harassment in the Workplace.

[8] Stanley K.M., Martin M.M., Nemeth L.S., Michel Y., Welton J.M. (2007). Examining lateral violence in the nursing workforce. Issues Ment. Health Nurs..

[9] Magnavita N., Meraglia I. (2024). Poor Work Ability Is Associated with Workplace Violence in Nurses: A Two-Wave Panel Data Analysis. Int. J. Environ. Res. Public Health.

[10] Bamberger E., Bamberger P. (2022). Unacceptable behaviours between healthcare workers: Just the tip of the patient safety iceberg. BMJ Qual. Saf..

[11] Guo L., Ryan B., Leditschke I.A., Haines K.J., Cook K., Eriksson L., Olusanya O., Selak T., Shekar K., Ramanan M. (2022). Impact of unacceptable behaviour between healthcare workers on clinical performance and patient outcomes: A systematic review. BMJ Qual. Saf..

[12] Gale J., Erez A., Bamberger P., Foulk T., Cooper B., Riskin A., Schilpzand P., Vashdi D. (2024). Rudeness and team performance: Adverse effects via member social value orientation and coordinative team processes. J. Appl. Psychol..

[13] Zhang Y., Yin R., Lu J., Cai J., Wang H., Shi X., Mao L. (2022). Association between horizontal violence and turnover intention in nurses: A systematic review and meta-analysis. Front. Public Health.

[14] Al-Nawafleh A.H., Al-Hamdan Z.M., Bawayzah H., Bawadi H. (2024). The influence of horizontal violence on intention to leave among Jordanian nurses: A cross-sectional study. PLoS ONE.

[15] Layne D., Beall C., Bryant W.T., Morris L., Craven H. (2024). Experiences with Negative Behavior and Incivility: Perspectives of Unlicensed Assistive Personnel and Registered Nurses. Nurs. Rep..

[16] Xie Q., Xu H., Luo Z., Gong A., Wang L., Zhou J. (2024). Influencing factors of inter-nursing lateral violence: A qualitative systematic review. J. Clin. Nurs..

[17] Alshawush K., Hallett N., Bradbury-Jones C. (2022). The impact of transition programmes on workplace bullying, violence, stress and resilience for students and new graduate nurses: A scoping review. J. Clin. Nurs..

[18] Einarsen S.V., Hoel H., Zapf D., Cooper C.L. (2020). The Concept of Bullying and Harassment at Work: The European Tradition. Bullying and Harassment in the Workplace.

[19] World Health Organization (2020). State of the World’s Nursing 2020: Investing in Education, Jobs and Leadership.

[20] Kurtovic B., Civka K., Cukljek S., Boskovic S., Kovacevic I., Spevan M., Brusic J., Friganovic A. (2024). A Single-Centre Study of Factors Associated with Components of Burnout Among Nursing Students in Croatia. Int. J. Crit. Care.

[21] Friganović A., Selič P. (2021). Where to Look for a Remedy? Burnout Syndrome and its Associations with Coping and Job Satisfaction in Critical Care Nurses—A Cross-Sectional Study. Int. J. Environ. Res. Public Health.

[22] Vukša A., Bušac V., Žepina Puzić A. (2023). Nasilje među medicinskim sestrama Violence among nurses. Sestrin. Glas. J..

[23] Friganović A., Slijepčević J., Režić S., Alfonso-Arias C., Borzuchowska M., Constantinescu-Dobra A., Coțiu M.-A., Curado-Santos E., Dobrowolska B., AGutysz-Wojnicka A. (2024). Critical Care Nurses’ Perceptions of Abuse and Its Impact on Healthy Work Environments in Five European Countries: A Cross-Sectional Study. Int. J. Public Health.

[24] Grozdek D. (2022). Učestalost Nasilja Nad Medicinskim Sestrama/Tehničarima u Zdravstvenim Ustanovama. Ph.D. Thesis.

[25] Layne D.M., Nemeth L.S., Mueller M., Wallston K.A. (2019). The Negative Behaviors in Healthcare Survey: Instrument Development and Validation. J. Nurs. Meas..

[26] (2018). HKMS. Nasilje nad Medicinskim Sestrama i Sigurnost u Zdravstvenim Ustanovama. Plavi Fokus.

[27] Polit D.F., Yang F.M. (2016). Measurement and the Measurement of Change: A Primer for the Health Professions.

[28] Spreng R.N., McKinnon M.C., Mar R.A., Levine B. (2009). The Toronto Empathy Questionnaire. J. Pers. Assess..

[29] Buss A.H., Perry M. (1992). The Aggression Questionnaire. J. Pers. Soc. Psychol..

[30] Field A. (2013). Discovering Statistics Using IBM SPSS Statistics.

[31] Layne D.M., Nemeth L.S., Mueller M. (2020). Negative Behavior Among Healthcare Professionals: Integrative Review of Instruments. J. Nurs. Meas..

[32] Joshi A., Kale S., Chandel S., Pal D.K. (2015). Likert Scale: Explored and Explained. Curr. J. Appl. Sci. Technol..

[33] Anderson C.A., Bushman B.J. (2002). Human aggression. Annu. Rev. Psychol..

[34] Bentler P.M. (1992). On the fit of models to covariances and methodology to the Bulletin. Psychol. Bull..

[35] Hu L., Bentler P.M. (1999). Cutoff criteria for fit indexes in covariance structure analysis: Conventional criteria versus new alternatives. Struct. Equ. Model..

[36] Browne M.W., Cudeck R. (1993). Testing Structural Equation Models.

[37] Mueller R.O., Mueller R.O. (1996). Confirmatory Factor Analysis. Basic Principles of Structural Equation Modeling: An Introduction to LISREL and EQS.

[38] Jung S., Lee H.-J., Lee M.Y., Kim E.S., Jeon S.-W., Shin D.-W., Shin Y.-C., Oh K.-S., Kim M.-K., Cho S.J. (2023). Gender Differences in the Association between Workplace Bullying and Depression among Korean Employees. Brain Sci..

[39] Luca C.E., Sartorio A., Bonetti L., Bianchi M. (2024). Interventions for Preventing and Resolving Bullying in Nursing: A Scoping Review. Healthcare.

[40] Chachula K., Ahmad N., Smith N., Henriquez N. (2022). Incivility in Nursing Education: Sources of Bullying and their Impact on Nursing and Psychiatric Nursing Students. Qual. Adv. Nurs. Educ. Avancées Form. Infirm..

[41] Mohammed Al Mansoor K. (2024). Relation between Workplace Bullying and Staff Nurses Self-Efficacy: A Cross-Sectional Study. Egypt. J. Health Care.

[42] Xu H., Xue M., Takashi E., Kitayama A., Zhu P., Liu Y. (2022). Exploring the relationship between lateral violence and nursing professionalism through the mediating effect of professional identity: A cross-sectional questionnaire study. Nurs. Open.

